# Larvicidal Activity against *Aedes aegypti* and Molluscicidal Activity against *Biomphalaria glabrata* of Brazilian Marine Algae

**DOI:** 10.1155/2014/501328

**Published:** 2014-02-13

**Authors:** Elíca Amara Cecília Guedes, Cenira M. de Carvalho, Karlos Antonio Lisboa Ribeiro Junior, Thyago Fernando Lisboa Ribeiro, Lurdiana Dayse de Barros, Maria Raquel Ferreira de Lima, Flávia de Barros Prado Moura, Antônio Euzebio Goulart Sant'Ana

**Affiliations:** ^1^Laboratório de Produtos Naturais, Instituto de Química e Biotecnologia, Universidade Federal de Alagoas, Campus A., C. Simões, Avenida Lourival Melo Mota, s/n, Tabuleiro do Martins, 57072-970 Maceió, AL, Brazil; ^2^Universidade Federal de Alagoas, Icbs Campus A., C. Simões, Avenida Lourival Melo Mota, s/n, Tabuleiro do Martins, 57072-970 Maceió, AL, Brazil

## Abstract

This study investigated the biological activities of five benthic marine algae collected from Northeastern Region of Brazil. The tested activities included larvicidal activity against *Aedes aegypti*, molluscicidal activity against *Biomphalaria glabrata,* and toxicity against *Artemia salina*. Extracts of *Ulva lactuca* (Chlorophyta), *Padina gymnospora, Sargassum vulgare* (Phaeophyta), *Hypnea musciformis,* and *Digenea simplex* (Rhodophyta) were prepared using different solvents of increasing polarity, including dichloromethane, methanol, ethanol, and water. Of the extracts screened, the dichloromethane extracts of *H. musciformis* and *P. gymnospora* exhibited the highest activities and were subjected to bioassay-guided fractionation in hexane and chloroform. The chloroform fractions of the *P. gymnospora* and *H. musciformis* extracts showed molluscicidal activity at values below 40 **μ**g*·*mL^−1^ (11.1460 **μ**g*·*mL^−1^ and 25.8689 **μ**g*·*mL^−1^, resp.), and the chloroform and hexane fractions of *P. gymnospora* showed larvicidal activity at values below 40 **μ**g*·*mL^−1^ (29.018 **μ**g*·*mL^−1^ and 17.230 **μ**g*·*mL^−1^, resp.). The crude extracts were not toxic to *A. salina*, whereas the chloroform and hexane fractions of *P. gymnospora* (788.277 **μ**g*·*mL^−1^ and 706.990 **μ**g*·*mL^−1^) showed moderate toxicity, indicating that the toxic compounds present in these algae are nonpolar.

## 1. Introduction

Marine environments, particularly in the tropics, have greater species richness than tropical forests. These diverse species are capable of producing a wide variety of chemical compounds with unique structures and functions, and many of these compounds can be used for the development of novel drugs [1, 2]. As an abundant resource in Brazilian coastal waters, marine algae exhibit a great potential source of novel compounds. It is known that species of marine algae synthesize bioactive compounds with diverse activities [2]. Many studies aiming to identify representative genera of marine algae that produce bioactive substances have been conducted. In the last three decades, the rate of discovery of biologically active metabolites produced by macroalgae has increased [3–5].

The secondary metabolites of marine algae are analogous to those of terrestrial plants with respect to the production of terpenes, aromatic compounds, acetogenins, amino acid derivatives, and polyphenols [5]. Many of these metabolites are known to possess antibacterial, antifungal, antiviral, and antitumor activities [1].

The Brazilian Northeast Region is the poorest region of Brazil and has the worst Human Development Indices in the country [6]. Most of the population in this region is at risk of contracting neglected tropical diseases. In particular, dengue fever and schistosomiasis are major public health concerns in Brazil and other developing tropical countries [7, 8]. Natural products with different biocidal activities can help fight parasite vectors at the adult or larval stages and are good alternatives to synthetic products due to their rapid biodegradation and lower costs [9], thus contributing to improved public health conditions in tropical developing countries.

The present study investigates the biological activities of five marine algae: *Ulva lactuca* Linnaeus, *Padina gymnospora *(Kutzing) Sonder, *Sargassum vulgare* C. Agardh, *Hypnea musciformis *(Wulfen in Jacque) J.V. Lamouroux, and *Digenea simplex* (Wulfen) C. Agardh—all collected from the coast of Alagoas State. The activities tested included larvicidal activity against* Aedes aegypti,* molluscicidal activity against *Biomphalaria glabrata,* and toxicity against *Artemia salina.* This is the first report on the biological activity of marine algae collected from the coast of Alagoas.

## 2. Materials and Methods

### 2.1. Collection of Marine Algae

Algae were manually collected from the Riacho Doce beach (9°34′0′′S and 35°39′0′′W) during low tides between October 2007 and July 2009. The collected samples were immediately transported to the Phycology Laboratory of the Biological Sciences Institute (ICBS), Universidade Federal de Alagoas (UFAL). Five species of algae from three divisions were used for this study: Chlorophyta—*Ulva lactuca* Linnaeus, MAC51238; Phaeophyta—*Padina gymnospora *(Kutzing) Sonder, MAC51235, and *Sargassum vulgare* C. Agardh, MAC51236; and Rhodophyta—*Hypnea musciformis *(Wulfen in Jacque) J.V. Lamouroux, MAC51234, and *Digenea simplex* (Wulfen) C. Agardh, MAC51231. A voucher specimen of each algae species was deposited in the MAC Herbarium at the Environmental Institute of the city of Maceió, Alagoas (Br), as a reference material.

### 2.2. Extract Preparation and Fractionation

Algae were washed with distilled water, dried in air-circulating ovens (BLUE, MOD-1401440SC, USA) at 45°C for three hours, and crushed using a MetVisa industrial blender (model type TA-2). The dried and crushed material from each collection and each alga was mixed in equal quantities. To obtain crude extracts, 500 g of dried algae was suspended in 1000 mL of dichloromethane, methanol, ethanol, or water and macerated for 72 hours; this process was repeated three times. The organic extracts were filtered and rotoevaporated (MOD Rotoevaporator with a Buchi heating bath B-490, Switzerland) at 25°C–40°C, and the aqueous extract was lyophilized (Edwards Freeze dryer Mod E2 MB of Brazil). Extracts of the respective algal species were stored at 4°C. The dichloromethane extracts of *H. musciformis* and *P. gymnospora *were selected for fractionation because they gave the best yields: 11.86 g (2.58%) and 13.96 g (3.04%), respectively. The yields of the other extracts were less than 0.5 g (0.1%) and were therefore not sufficiently large for fractionation.

For fractionation by liquid-liquid partitioning, the crude extracts of *H. musciformis* (2.73 g) and *P. gymnospora* (1.90 g) were suspended in methanol : water (3 : 1) and extracted with hexane and chloroform to yield chloroform and hexane fractions. All solvents were purchased from VERTEC Fine Chemicals of Brazil.

### 2.3. Mosquito Larvicidal Activity

Bioassays were run with *A. aegypti* larvae at the Chemistry and Biotechnology Institute of Federal University from Alagoas IQB/UFAL laboratory using the WHO protocol [10]. The bioassays used fourth instar larvae with two repetitions. Each experimental course used 10 larvae placed in a 250 mL cup containing 27 mL of test solution at 100 *μ*g·mL^−1^. Extracts that led to ≤50% mortality were subjected to more precise tests. In these tests, four replicates were run using 25 larvae in each replicate. The concentrations used were defined based on the preliminary results.

Larvae were considered dead when they failed to reach the surface of the solution after the liquid was stirred. Dead larvae counts were performed at 24 hours and 48 hours after the start of the experiment, and the samples were run in quadruplicate. In parallel, control tests were performed with 1% aqueous dimethyl sulfoxide (DMSO) solution and the synthetic larvicide Temephos at 3 *μ*g·mL^−1^. The activity level of extracts tested was calculated based on the average percent mortality of larvae at 48 hours (>75%: highly active, 50–75%: moderately active, 25–50%: weakly active, and <25%: inactive).

For *A. aegypti *larvae, treatments that result in at least 50% mortality within 48 h were selected for subsequent bioassays using different concentrations to determine the concentrations required to kill 10% (LC_10_), 50% (LC_50_), and 90% (LC_90_) of the larvae. Analysis was performed according to the probit method [11].

### 2.4. Toxicity Test with *Artemia salina* (Nontarget Model Organism)

*Artemia salina* toxicity (i.e., LC_50_) is largely accepted [12] as a parameter reflecting the general biological toxicity of a compound in nontarget organisms. Toxicity tests using *Artemia salina* Leach nauplii were carried out according to the method described by Bastos and colleagues [13], with some modifications. *Artemia* cysts (approximately 1 g·L^−1^) were added to filtered seawater and maintained at 27–30°C with aeration and under continuous light. Samples that showed ≥30% mortality at 1000 mg·mL^−1^ in preliminary tests were subjected to quantitative assays. After 24 hours, the nauplii hatched, were collected with a micropipette, and were concentrated in a Petri dish with seawater maintained at the same temperature and luminosity for more than 24 hours. Fifteen grams of each crude extract (dichloromethane, methanol, ethanol, and water) was diluted in 5 mL of distilled water. The control solution was prepared with seawater and 0.1% dimethyl sulfoxide (DMSO).

Ten *Artemia* nauplii and 50 *μ*L algae extract were placed in each well. Four different concentrations of each extract were tested in six replicates. A control solution was also tested in six replicates. After 24 hours, the number of dead larvae was determined with the aid of a Zoom stereomicroscope. The dead larvae counts also included larvae that remained immobile after stimulation for at least 10 seconds.

The degree of toxicity of each plant extract fraction was determined as recommended by Déciga-Campos et al. [14]. These authors consider extracts that have an LC_50_ ≥ 1000 *μ*g·mL^−1^ to be nontoxic, an LC_50_ between 500 and 1000 *μ*g·mL^−1^ to be weakly toxic, and LC_50_ < 500 *μ*g·mL^−1^ to be highly toxic. The LC_50_ values (concentrations that cause 50% mortality) and their confidence intervals (90% IC and 95% IC) were calculated using probit analysis [11].

### 2.5. Molluscicidal Activity

Molluscicidal activity was tested in *Biomphalaria glabrata* (Say 1818) that were propagated in the laboratory and were descendants of specimens not infected by trematodes.

Snails were kept in glass tanks at room temperature in a dechlorinated water system for 8 hours with a mixture of soil, sand, and calcium carbonate. During the assay period, they were fed fresh lettuce and fish food. Molluscicidal activity was evaluated by immersing *B. glabrata* in an aqueous solution mixed with 0.1% dimethyl sulfoxide (DMSO) containing the extract under investigation at different concentrations (0.10 to 100 *μ*g·mL^−1^) [8].

The test solution for each algal extract was prepared at 100 *μ*g·mL^−1^ for the preliminary tests with the snails. In the preliminary stage, five adult snails were used per concentration. Two sets of controls were used to verify that the snails had normal susceptibility to toxins. The positive control used was niclosamide at 3 *μ*g·mL^−1^ and the negative control was dechlorinated water. The organisms were exposed to the extracts for 24 hours and observed for 72 hours, with readings and water exchanges every 24 hours. Dead specimens were removed at the time of the readings. The death of the snails was indicated by discoloration, the absence of muscle contractions, bleeding, and the deterioration of body tissues. The algal extract promoted approximately 30% mortality at 100 *μ*g·mL^−1^. In parallel, control tests were performed with aqueous DMSO solution (0.1%) and the molluscicide niclosamide at 3 *μ*g·mL^−1^. Probit version 1.5 [11] was used to calculate the lethal concentration values (LC_90_, LC_50_, and LC_10_). The World Health Organization (WHO) suggests that crude extracts of plants with LC_50_ values <40 *μ*g·mL^−1^ (i.e., 0.04% or 0.4 mg·mL^−1^) have some potential as molluscicidal or larvicidal compounds [10].

The results shown for the molluscicidal tests represent the average ± standard deviation of three replicates (*n* = 3). These results were submitted to ANOVA, followed by the Tukey test (*P* ≤ 0.05). All tests were performed using the Microcal Origin, version 8.0, and Graph Pad Prism, version 5.0.

## 3. Results and Discussion

The crude dichloromethane, methanol, ethanol, and water extracts from different algal species (*Ulva lactuca* (Chlorophyta), *Padina gymnospora*, *Sargassum vulgare* (Phaeophyta), *Hypnea musciformis,* and *Digenea simplex *(Rhodophyta)) and the chloroform and hexane fractions of extracts from *H. musciformis* and* P. gymnospora* were screened for biological activity. Different concentrations of extracts were tested according to organism susceptibility. The initial concentrations used were 1000 *μ*g·mL^−1^ for the *Artemia salina* toxicity test, 500 *μ*g·mL^−1^ for the *Aedes aegypti* larvicidal test, and 100 *μ*g·mL^−1^ for the *Biomphalaria glabrata* molluscicidal test.

The *A. aegypti* larvicidal tests revealed that only the hexane and chloroform fractions of *P. gymnospora* exhibited larvicidal activity, with LC_50_ values of 29.01 *μ*g·mL^−1^ and 17.23 *μ*g·mL^−1^, respectively (Table 1); the other extracts exhibited lower activities.

Selvin and Lipton [9] evaluated the biocidal potential of *Ulva fasciata *and *H. musciformis *isolated in India and verified their larvicidal activity against the mosquito *Culex *sp. at the second and fourth instars. These authors found that larvae at second instar exhibited 100% mortality when exposed to 10 mg·mL^−1^ methanol : dichloromethane extracts (1 : 1). Although the mosquito species used in our study was not the same as that used by those authors, both *Culex* sp. and *A. aegypti* are vectors of viral diseases, and extracts from *H. musciformis* showed no lethality against *A. aegypti*. An analysis of the larvicidal activities of methanol extracts of 20 species of seaweed collected off the coast of India against the second instar larvae of *A. aegypti *[2] revealed that *Lobophora variegata* (Phaeophyta-Dictyotaceae) had a relatively high LC_50_ (70.38 *μ*g·mL^−1^). In our work, only the hexane and chloroform fractions of *P. gymnospora* (Phaeophyta) had higher larvicidal potentials (29.018 *μ*g·mL^−1^ and 17.230 *μ*g·mL^−1^, resp.).

The chloroform fractions from *H. musciformis and P. gymnospora* showed molluscicidal activity against *B. glabrata,* and their LC_50_ values were determined to be 11.1460 and 25.8689 *μ*g·mL^−1^, respectively. The hexane fractions of these two algal species did not show molluscicidal activity sufficient to calculate a lethal concentration (Table 2).

Given the molluscicidal activity of the chloroform fractions of *H. musciformis *and* P. gymnospora*, and the fact that the WHO [10] recommends that plant extracts with LC_50_ < 40 *μ*g·mL^−1^ be used directly against *B. glabrata*, these fractions might be promising sources of new compounds with molluscicidal activity. Patel et al. [15] evaluated the molluscicidal potential of methanolic extracts from 60 marine algae and found that only extracts from *Fucus serratus*, *F. vesiculosus*, *Pelvetia canaliculata*, *Ascophyllum nodosum*, *Halidrys siliquosa*, *Bifurcaria bifurcata*, *Dictyota dichotoma, *and *Halopithys incurve* exhibited activity against *B. glabrata *at 500 *μ*g·mL^−1^.

*A. salina* toxicity upon exposure to crude dichloromethane, methanol, ethanol, and water extracts and to the chloroform and hexane fractions of algae *H. musciformis and P. gymnospora *was analyzed. No mortality was observed at any concentration tested for any samples, except the chloroform and hexane fractions of algae *H. musciformis and P. gymnospora*; thus, the LC values could not be calculated.

The LC_50_ value reflects the concentration necessary to cause the death of 50% of the *A. salina* individuals in a sample. Therefore, lower LC_50_ values reflect greater toxicity [16]. The results of the *A. salina* assay indicated that crude extracts of *P. gymnospora* prepared with dichloromethane, methanol, and ethanol and crude extracts of *H. musciformis* prepared with dichloromethane, methanol, and water had low toxicities (Table 3). The chloroform and hexane fractions of the dichloromethane extract of *P. gymnospora* showed low toxicity against *A. salina* (lower than 1000 *μ*g·mL-1) (Table 4). For the other marine algae extracts tested, no LC could be calculated.

Ara et al. [17] investigated the toxic activity of ethanol extracts of 22 algal species from the Karachi coast against *A. salina *and found that five brown algae showed values below 1000 *μ*g·mL^−1^ (*Spatoglossum asperum*, *Stokeyia indica *(507 *μ*g·mL^−1^), *Stoechospermum marginatum* (612 *μ*g·mL^−1^ *), Sargassum swartzii* (928 *μ*g·mL^−1^), and *S. binderi* (735 *μ*g·mL^−1^)), whereas only one green alga *Caulerpa racemosa* (929 *μ*g·mL^−1^) was toxic. In the same study, these authors found that the hexane fractions of *S. marginatum *(349 *μ*g·mL^−1^) and *S. swartzii* (61 *μ*g·mL^−1^) were also toxic. In our study, the chloroform and hexane fractions of *P. gymnospora* (788.277 *μ*g·mL^−1^ and 706.990 *μ*g·mL^−1^, resp.) showed low activity, indicating the polar nature of the toxic compounds of this alga.

Lhuillier et al. [18] screened 26 benthic macroalgae in the state of Santa Catarina and assayed their lethality using *A. salina* larvae; the samples included ethanol extracts from red (Rhodophyta), brown (Phaeophyta), and green (Chlorophyta) algae. These authors found that 25 species had significant toxicity, especially *Acanthophora spicifera, Galaxaura marginata, Gracilaria dominguensis, Pterocladia capillacea*, *Padina gymnospora*, *Ectocarpus breviarticulatus, Chaetomorpha antennina, *and *Codium decorticatum *(50 *μ*g·mL^−1^). In the present study, the ethanol extract of *H. musciformis* was not sufficiently lethal to allow the calculation of the LC_50_, and* P. gymnospora* was not lethal. Guedes et al. [19] found that fractions of *H. musciformis* and *P. gymnospora* have a selective cytotoxic activity against cancerous cells.

The generally low toxicity of algal extracts against *A. salina*, as reported by these authors, is consistent with the findings of this study. These results indicate that these extracts have some potential for the development of products to control vectors of neglected diseases without any important impacts on nontarget organisms, that is, organisms that could be inadvertently affected by toxic substances as a result of sharing an environment or resources with the target species.

## 4. Conclusions

The biological activity of two marine algae collected from the coast of Alagoas State showed larvicidal activity against* Aedes aegypti* (based on *Aedes aegypti* larvae in the fourth instar) and molluscicidal activity against *Biomphalaria glabrata *(based on the snail). The chloroform fractions of *H. musciformis *and* P. gymnospora* might be promising sources of new compounds with molluscicidal activity, and the hexane and chloroform fractions of *P. gymnospora* could be sources of novel larvicides.

The primary advantage of using these extracts would be their low cost of production, as the algae from which they were obtained are an abundant resource on the Brazilian coast. These algae are easy to cultivate, and their crude extracts have low off-target toxicity, as demonstrated in the *Artemia *nauplii.

Therefore, we suggest the extension of studies to establish the potential of these extracts for use in natural environments, considering the potential low impact on nontarget organisms.

## Figures and Tables

**Table 1:** Lethal concentrations (LC_50_) of the chloroform and hexane fractions (in *μ*g·mL^−1^) of *P. gymnospora* for *Aedes aegypti*.

Algae/fraction	LC_10_	LC_50_	LC_90_
C_6_H_14_ (a)	3.004 ≥ 7.67 ≤ 12.373	29.018 ≤ 39.155 ≥ 54.584	117.977 ≥ 199.791 ≤ 572.243
CHCL_3_ (b)	0.992 ≥ 3.009 ≤ 5.559	17.230 ≤ 25.340 ≥ 37.063	116.650 ≥ 213.374 ≤ 633.942

(a) Hexane fraction; (b) chloroform fraction.

**Table 2:** Lethal concentration (LC_50_) of the hexane (C_6_H_14_) and chloroform (CHCL_3_) fractions of extracts from marine algae species collected on the beach of Riacho Doce, AL, against *B. glabrata. *

Algae/extract	LC_10_ (*μ*g·mL^−1^)	LC_50 _(*μ*g·mL^−1^)	LC_90 _(*μ*g·mL^−1^)
	(95% CI)	(95% CI)	(95% CI)
*H. musciformis*/**CH** **C** **L**_3_	2.7831 (2.2906–3.2838)	11.1460 (10.1599–12.161)	44.6382 (39.5253–51.3482)
*P. gymnospora*/**CH** **C** **L**_3_	5.963 (4.8189–7.1286)	25.8689 (23.4123–28.4624)	112.2189 (96.8318–133.4954
H. musciformis*/**C**_6_ **H**_14_	—	—	—
P. gymnospora*/**C**_6_ **H**_14_	—	—	—

*The LC could not be calculated.

**Values in parentheses correspond to the confidence intervals.

**Table 3:** Accurate toxicity test with crude extracts of marine algae collected on the beach Riacho Doce, AL, against *A. salina* (CH_2_Cl_2_: dichloromethane; MeOH: methanol; EtOH: ethanol; H_2_O: water).

Algae/extract	LC_10_ (*μ*g·mL^−1^)	LC_50_ (*μ*g·mL^−1^)	LC_90_ (*μ*g·mL^−1^)
*H. musciformis*/CH_2_CL_2_	540.54 ( 410.67–620.21)	1070.90 (930.20–1480.02)	2130.46 (150.039–4780.61)
*P. gymnospora* CH_2_CL_2_	440.56 (250.32–550.61)	1160.01 (940.48–2130.66)	2950.41 (1770.11–16330.47)
*H. musciformis*/MeOH	590.73 (470.908–670.167)	1130.02 (970.18–1570.13)	2130.85 (1540.77–4680.26)
*P. gymnospora*/MeOH	640.42 (560.31–700.235)	1000.78 (901.74–1170.73)	1570.69 (1300.87–2250.43)
*P. gymnospora/*EtOH	830.84 (720.39–970.64)	1430.59 (1150.01–3070.08)	2450.94 (1630.69–10770.90)
*H. musciformis/*H_2_O	550.25 (310.95–650.74)	1450.61 (1080.68–476.40)	3830.69 (2000.35–6360.94)

Values in parentheses correspond to the confidence intervals.

**Table 4:** More precise toxicity test of the hexane (C_6_H_14_) and chloroform (CHCl_3_) fractions of the extracts of algal species collected on the beach Riacho Doce, AL, against *A. salina. *

Algae/extract	LC_10_ (µg·mL^−1^)	LC_50_ (µg·mL^−1^)	LC_90_ (µg·mL^−1^)
*P. gymnospora * **C**_6_ **H**_14_	330.474 (139.004–437.601)	788.277 (687.856–998.205)	1880.271 (1307.848–5926.118)
*H. musciformis **C**_6_ **H**_14_	—	—	—
*P. gymnospora * **C** **H** **C** **L**_3_	423.49 (322.234–489.074)	706.990 (648.747–776.152)	1180.111 (1009.600–1593.490)
*H. musciformis **C** **H** **C** **L**_3_	—	—	—

*The LC could not be calculated; values in parentheses correspond to the confidence intervals.
